# Supplementary material for: Feasibility Study of an Internet-Based Platform for Tele-Neuropsychological Assessment of Elderly in Remote Areas
Source: Diagnostics (Basel). 2022 Apr 7;12(4):925. doi: 10.3390/diagnostics12040925 (PMC9031122; doi:10.3390/diagnostics12040925)
Supplement: Supplementary file 1 [file diagnostics-12-00925-s001.zip › diagnostics-1630575-supplementary.pdf]

Évaluation de l'expérience utilisateur

Questionnaire sur l'acceptation de la procédure à distance pour les tests cognitifs

Consigne : Merci de répondre à ce questionnaire. **Pour répondre, veuillez cocher la case correspondant à votre choix.**

1. Je suis globalement satisfait de cette expérience.

Complètement en désaccord Complètement d'accord

1	2	3	4	5	6	7

Commentaires :

____

____

____

2. Globalement, le système est facile à utiliser.

Complètement en désaccord Complètement d'accord

1	2	3	4	5	6	7

Commentaires :

____

____

____

3. Les instructions étaient claires et compréhensibles.

Complètement en désaccord Complètement d'accord

1	2	3	4	5	6	7

Commentaires :

____

____

____

4. Je répéterai cette expérience.

Complètement en désaccord Complètement d'accord

1	2	3	4	5	6	7

Commentaires :

___

___

___

5. Avez-vous envisagé de vous retirer de l'étude ou de l'expérience à un moment donné ?

**OUI**	**NON**

Commentaires :

___

___

___

6. Quelle méthode d'évaluation préférez-vous, en face à face ou par vidéoconférence ?

**face à face**	**vidéoconférence**

7. Sur une échelle de zéro à dix, quelle est le degré de probabilité pour que vous recommandiez cette méthode d'évaluation à un ami ou un collègue ?

Complètement en désaccord Complètement d’accord

**1**	**2**	**3**	**4**	**5**	**6**	**7**	**8**	**9**	**10**

8. Qu'est-ce qui manquait ou qui vous a déçu durant votre expérience ?

___

___

9. Qu'est-ce que vous avez le plus/le moins aimé dans cette procédure ?

___

___

10. Quel serait LE moyen pour améliorer le système ?

___

___
